# Ephemerality in Social Media: Unpacking the Personal and Social Characteristics of Time Limit Users on WeChat Moments

**DOI:** 10.3389/fpsyg.2021.712440

**Published:** 2021-09-06

**Authors:** Yun Zhang, Hongyan Wang, Chuan Luo, Siyu Chen

**Affiliations:** ^1^School of Economic Information and Engineering, Southwestern University of Finance and Economics, Chengdu, China; ^2^The Collaborative Innovation Centre of the Innovation and Regulation of Internet-Based Finance, Southwestern University of Finance and Economics, Chengdu, China; ^3^School of Business Administration, Southwestern University of Finance and Economics, Chengdu, China

**Keywords:** ephemerality, Time Limit, WeChat Moments, personal characteristics, social characteristics, users and nonusers

## Abstract

Social media platforms increasingly give users the option of ephemerality through settings that delete or hide posted content after a set period of time. Many individuals apply these settings to manage their posting history and, in turn, reduce concerns about self-presentation. Despite the growing popularity of this feature, few studies have empirically explored it. This study examines the Time Limit setting on WeChat Moments as an example and investigates how users using the Time Limit setting differ from nonusers in terms of personal characteristics (demographics, personality traits, psychological factors, and previous behavioral patterns) and social characteristics (audience size and audience diversity). Compared with nonusers, users using Time Limit setting scored significantly higher on posting frequency and privacy setting use and scored significantly lower on audience size. We also examine how personal and social characteristics vary between user groups with different degrees of ephemerality (i.e., low, medium, or high). Our findings show that users using the Time Limit setting who scored higher on measures of life changes, self-monitoring, posting frequency, and audience size and lower on perceived stress were more likely to opt for the low (i.e., 6months) rather than the medium (i.e., 1month) or high (i.e., 3days) degree of ephemerality. Our work contributes to the understanding of ephemerality settings on social media platforms and provides insights that help practitioners design more effective platforms.

## Introduction

Most social media platforms keep past posts online indefinitely to help users with their long-term self-presentation and interactions with others (Zhao et al., 2013; Özkul and Humphreys, 2015). However, this feature may also create challenges for users when past posts are inconsistent with their current self-presentation (Schoenebeck et al., 2016; Huang et al., 2020). As such, it is becoming increasingly common for social media platforms to allow users to make their posted content ephemeral using relevant settings (Xu et al., 2016; Chen and Cheung, 2019). For instance, on Snapchat Stories and Instagram Stories, users’ posted content disappears after 24h. Most users of this feature report fewer privacy concerns and perceive greater enjoyment when using Snapchat (Morlok et al., 2017; Choi and Sung, 2018). Nonetheless, they also experience certain types of loss such as media loss (e.g., the failure to save photographs) or context loss (e.g., the lack of a message history; Cavalcanti et al., 2017).

Likewise, WeChat Moments, one of the most popular social media platforms in China, provides a Time Limit option, which allows users to set a time span (3days, 1month, or 6months) for the visibility of their posts, after which their content expires and becomes viewable only by the posters themselves. A report from January 2021 (Tencent, 2021) states that more than 200 million users make use of the “Time Limit” feature. Despite the fact that it can, in certain circumstances, have negative effects such as undermining social relationships when contacts have a feeling that the person using the feature does not trust them (Li et al., 2018), it can also have positive effects by supporting users’ evolving self-presentation (Huang et al., 2020). Zheng and Zhao (2020) identified 16 factors that influence the “Time Limit” usage: influence of old and new relation, information changes, self-recording, etc. However, research on the Time Limit usage is still in its infancy, and further investigation is needed (e.g., in profiling users using this feature).

Investigating the differences between users and nonusers of ephemerality settings can lead to an understanding of individuals’ adoption behaviors. It can also provide insight into how relevant settings on social media platforms can be improved. Prior research has indicated that personal characteristics (e.g., demographics, personality traits) are the main factors that influence innovation–adoption behaviors and social media use (Hargittai, 2007; Meng et al., 2015; Brailovskaia and Margraf, 2016; Scott et al., 2020). Also, social characteristics (e.g., the number of intimate friendships) were found to affect users’ attitudes and behaviors on social media (Ljepava et al., 2013; Grieve, 2017). This study examines the differences between users and nonusers of ephemerality settings by focusing on their personal and social characteristics, using the Time Limit setting on WeChat Moments as an example. Building on relevant research on the use of privacy settings in social media (Vitak, 2012; Litt, 2013; Stern and Salb, 2015; Li et al., 2018; Ran et al., 2020), we focus on personal characteristics related to demographics (i.e., age, gender, education, and life change experiences), personality traits (i.e., self-esteem, self-monitoring, and emotional stability), psychological factors (i.e., social anxiety, perceived stress, and loneliness), previous behavior patterns (i.e., posting frequency and privacy setting use), and social characteristics (i.e., audience size and audience diversity). Time Limit users can also be categorized into three groups based on the degree of ephemerality they adopt: those who opt for a low level of ephemerality (i.e., the 6-month option), those who opt for a medium level of ephemerality (i.e., the 1-month option) and those who choose a high level of ephemerality (i.e., the 3-day option). Accordingly, we also examine the differences between these Time Limit user groups. Our research questions are as follows.

RQ1: What are the differences between Time Limit users and nonusers in terms of their personal and social characteristics?
RQ2: How do personal and social characteristics vary between Time Limit user groups that have different degrees of ephemerality (i.e., low, medium, and high)?

Our work contributes to the literature in the following ways. First, it allows for a better understanding of the usage of ephemerality settings by revealing the characteristics of users and nonusers. Second, it highlights the roles that personal and social characteristics play in innovation adoption by comparing how these factors differ between the user and nonuser groups. Third, our work provides new insight into the usage of ephemerality settings by exploring the differences between user groups with different levels of ephemerality.

## Theoretical Background

### Ephemerality in Social Media

As social media posts accumulate, there is a growing worry among users that the long-term visibility of historical information may damage their self-image and pose a threat to future interactions (Zhao et al., 2013; Xu et al., 2016). As such, some scholars have claimed that it is essential to allow individuals to set an expiration date for their posts (Mayer-Schönberger, 2011; Ayalon and Toch, 2013). Some social media platforms (e.g., Snapchat, Instagram Stories, and WeChat Moments) allow users to make their posts ephemeral (Piwek and Joinson, 2016; McRoberts et al., 2017; Huang et al., 2020) – the posts are deleted or hidden after a specific period of time. Given the increased use of ephemerality settings on social media platforms, some scholars have begun to explore why individuals opt to make their content ephemeral and how this influences users’ attitudes and behaviors. For instance, Xu et al. (2016) explored the ephemerality feature on Snapchat and found that users could find values in ephemeral communication, such as reducing self-consciousness, preventing the accumulation of embarrassing content, and with less need to worry about unintended audiences. Also, the ephemerality could make users express authentic self (Choi and Sung, 2018; Choi et al., 2020) as well as support their evolving self-identity (Huang et al., 2020; Luria and Foulds, 2021). Likewise, Chen and Cheung (2019) investigated individuals’ motivations for using social media and found that the fear of missing out, trust, immediacy, and social pressure influence individuals’ feelings of gratification, which then facilitate their engagement with ephemeral content.

Nonetheless, the availability of ephemerality on social media platforms may also have negative effects. For instance, Cavalcanti et al. (2017) claimed that individuals who use ephemeral settings experience three types of losses: media loss, meaning loss, and context loss. Particularly, individuals lost their ability to display meaningful past posts (e.g., travel photos) to intended new friends for impression management. The use of ephemerality settings on social media platforms may also undermine social relationships because it may lead online contacts to feel that they are not trusted by the user, leading to feelings of being rejected (Li et al., 2018). The use of these settings may also create negative impressions among new friends, who may perceive the use of such settings as defensive, isolated, or aloof (Huang et al., 2020).

### Factors Influencing the Use of Privacy Settings on Social Media

Social media platforms provide users with various privacy settings to help them manage their audience and control their privacy (Chen and Marcus, 2012; Young and Quan-Haase, 2013). For instance, the Friend Lists feature on Facebook helps users segment their audience and direct information to particular people (Vitak, 2012). Research on the topic has explored the factors that affect individuals’ use of privacy settings on social media. Most found that the use of privacy settings was often associated with negative experiences such as privacy intrusion. Litt (2013) found that users with strong privacy concerns or who had undergone turbulent online experiences were more likely to use social media privacy tools. Research has also revealed that users’ personal characteristics (e.g., demographics, previous behavior patterns) affect their use of privacy settings. For instance, Brandtzæg et al. (2010) found that younger adults use more privacy settings on Facebook than do older adults. Stern and Salb (2015) suggested that individuals who frequently use social media are more likely to use privacy settings. Other scholars have found that some social characteristics (e.g., the quality of peer relationships) can also affect individuals’ use of social media privacy settings (Lewis et al., 2008; Vitak, 2012; Li et al., 2018). Vitak (2012) found that Facebook users with many and diverse online friends in their social networks are more likely to use the Facebook Friend Lists setting.

Although some scholars have explored which factors are associated with the use of privacy settings on social media platforms, most of this work has focused on general privacy settings, rather than a specific privacy setting. We aim to fill this gap by focusing on the Time Limit feature on WeChat Moments. Specifically, we compare the differences between Time Limit users and nonusers in terms of their personal and social characteristics. The findings from this study will advance the understanding of users’ attitudes and behaviors on social media platforms.

### The Time Limit Setting on WeChat Moments

WeChat Moments, a function of the instant messaging application WeChat, is one of the most popular social media platforms in China. It was reported that every day in January 2021, 780 million users viewed Moments and 120 million users shared a post on the platform (Tencent, 2021). WeChat Moments is similar to Facebook, but posts made on the platform are only viewable to people on users’ WeChat contact lists. To mitigate users’ concerns about past posts, Moments launched the Time Limit setting in 2017. The feature allows users to choose a time span for their posted content – 3days, 1month, or 6months – after which posted content is hidden from contacts and viewable only by the user (see Appendix A; if the “all” option is selected, then the users’ posts do not expire). When users employ the Time Limit setting, their profile pages display the notice “Only [time span] of Moments are visible” to their WeChat friends (see Appendix B). Once selected, the setting is universal in that it applies to all of the user’s posts and audiences until the setting is changed. In other words, the setting cannot be applied only to certain posts or a select audience.

Research on the Time Limit setting on Moments is very limited. To the best of our knowledge, only three studies have preliminarily explored it. Relying on interview data, Li et al. (2018) found that the use of the Time Limit setting undermined social relationships among users. In a separate study, which also relied on interviews, Huang et al. (2020) investigated how the Time Limit setting supports users’ evolving self-presentation and claimed that this setting could help users effortlessly manage their desired self-presentation as they matured. By conducting a text-mining analysis, Ran et al. (2020) found that audience management, mystery, emotional state, the intensity of use of other social networking services, peer influence, and life changes were factors that could possibly influence the use of the Time Limit setting.

## Methodology

### Participants and Procedure

The data for this study were collected through an online survey platform Sojump^1^ during December 2020 and January 2021, in China. Sojump is a professional online survey platform consists of 2.6 million members and more than 1 million people fill out questionnaires on this platform every day (Sojump, 2020). Participants will be randomly invited to join in a survey. We posted an advertisement on Sojump to recruit participants, and anyone who is interested in our survey could join us. To increase the response rate, we offered a reward of 5 yuan to 10 yuan to each participant. To set the screening criteria, participants were first asked whether they were users of WeChat. If they were not WeChat users, they did not need to fill out the rest of the questionnaire. If they were, they were asked questions about their use of the Time Limit setting on WeChat Moments, their personal characteristics (i.e., demographics, personality traits, psychological factors, and previous behavior patterns), and the characteristics of their social networks (i.e., audience size and audience diversity). We scrutinized the completed questionnaires and excluded responses from participants who gave duplicate answers, or who had completed the survey in an unrealistically short time (less than 2min). The final dataset comprised responses from 390 respondents. Most of the participants were female (56.4%), and most of them were 26–40years old (71.7%). The majority of the participants (77.7%) were company employees. Among them, 97 users set their WeChat Moments Time Limit to 3days, 101 users set it to 1month, and 65 users set it to 6months. A prior power analysis using G*Power (Faul et al., 2007) indicated that a sample size of *n*=70 Time Limit users and *n*=70 nonusers (total *N*=140) was required for power to be at 0.90 to reveal a medium effect, with alpha set at the 0.1 level (Greenwood et al., 2016; Schroeder and Cavanaugh, 2018). With *n*=263 Time Limit users and *n*=127 Time Limit nonusers, our sample satisfied the required size.

### Measurements

Unless otherwise noted, participants were asked to score each item on a five-point Likert scale (1=*strongly disagree* to 5=*strongly agree*). Based on the back-translation approach (Brislin, 1980), we translated the English measurements into Chinese by one English major graduate student, and then translated Chinese into English by an experienced professor. We compared them with the original English content and modified the inconsistencies. Then, we refined items further *via* feedback from two experts and four WeChat active users to improve the face validity of instruments.

#### Personal Characteristics

##### Demographics

Participants indicated their age (1=*20years or below*, 2=*21–25years*, 3=*26–30years*, 4=*31–40years*, 5=*41years and above*), gender (1=*male*, 2=*female*), and educational attainment (1=*junior high school or below*, 2=*high school degree*, 3=*college degree*, 4=*bachelor’s degree*, and 5=*master’s degree and above*). Life change experiences were measured using Ayalon and Toch’s (2017) three-item scale (*α*=0.839). Sample items included “Since publishing the post I have had major changes in my personal life (new relationship, new baby, moved to a new town or state, etc.)” and “Since publishing the post I have had major changes in my professional life (switched to a new job, finished college, etc.).”

##### Personality Traits

Self-esteem was measured using the 10-item scale adopted by Rosenberg (1965; *α* =0.918). Item 8, “I wish I could have more respect for myself,” was not suitable to the Chinese context and was deleted (Tian, 2006; Ding et al., 2017), leaving nine items. Sample items included “On the whole, I am satisfied with myself” and “All in all, I am inclined to feel that I am a failure (reverse).” Relying on previous literature (Child and Agyeman-Budu, 2010; Kauppinen-Raisanen et al., 2018), we adopted the first part of the self-monitoring scale (Lennox and Wolfe, 1984) to measure one’s ability to modify self-presentation (*α*=0.792). Sample items included “Once I know what the situation calls for, it is easy for me to regulate my actions accordingly” and “I have found that I can adjust my behavior to meet the requirements of any situation I find myself in.” The two-item subscale from the Ten-Item Personality Inventory (TIPI; Gosling et al., 2003) was used to measure emotional stability (*α*=0.766). Sample items included “I am anxious or easily upset (reverse)” and “I am calm or emotionally stable.”

##### Psychological Factors

We adopted the social anxiety subscale in the Self-Consciousness Scale (Fenigstein et al., 1975) to measure social anxiety (*α*=0.895). Sample items were “Large groups make me nervous” and “I have trouble working when someone is watching me.” Perceived stress was measured using the 14-item scale developed by Cohen et al. (1983; *α* =0.928). Sample items were “I often feel that I am unable to control the important things in my life” and “I often feel difficulties are piling up so high that I cannot overcome them (reverse).” Loneliness was measured using the 10-item abbreviated version of the UCLA Loneliness Scale (Russell, 1996; Reid and Reid, 2007; *α*=0.879). Participants were asked to report the quality of interpersonal relationships by responding to five positively and five negatively worded statements. Sample items were “How often do you feel you have nobody to talk to?” and “How often do you feel completely alone?”

##### Previous Behavior Patterns

The measure of posting frequency was adopted from Weiser’s (2015) research to measure the number of posts or user updates per month, using a five-point scale ranging from 1 (*once a month or less frequently*) to 5 (*eight times a month or more*). The use of privacy settings was measured by asking “How often do you use the tags when you share a post?” (1=*never use*, 2=*sometimes use*, 3=*frequently use*) as suggested by Vitak (2012). The tags feature is similar to Friend Lists on Facebook, which enables users to select audiences for a certain post, as shown in Appendix C.

#### Social Characteristics

Audience size was measured adopting the instrument developed by Lankton et al. (2017): we used a five-point scale ranging from 1 (*100 and below*) to 5 (*401 and above*) to measure the total size of each respondent’s WeChat audience. Audience diversity was measured using an approach similar to those of Vitak (2012) and Oeldorf-Hirsch et al. (2017). Participants were asked which types of online friends were part of their WeChat social networks: partner/spouse, friends, acquaintances, classmates, coworkers, family, boss, potential employers, teachers, strangers, and others. Audience diversity was calculated by taking the sum of the number of categories selected and using the number as the score (ranging from 1 to 11).

## Results

### Descriptive Statistics

Supplementary Table 1 shows the descriptive statistics and correlations between variables, as well as Supplementary Figures 1–3 shows frequency in age, gender, and education.

### Differences Between Time Limit Users and Nonusers

#### Differences in Personal Characteristics

To examine whether the Time Limit user and nonuser groups differed in terms of demographics, personality traits, psychological factors, and previous behavior patterns, we conducted independent sample *t* tests with the personal characteristics as dependent variables and the grouping variable (user vs. nonuser) as the factor. The scores for posting frequency and privacy setting use were treated as ordinals; thus, nonparametric group comparisons (Mann–Whitney U test) were conducted on these two variables. Gender was analyzed using Pearson chi-square tests (Gainsbury et al., 2016; shown in Supplementary Table 2). Moreover, we applied the false discovery rate (FDR; Benjamini and Hochberg, 1995) for multiple testing corrections with FDR-adjusted value of *p* reported. The results indicated that compared with nonusers, Time Limit users scored higher on education (*t*=1.85, *p*=0.066), posting frequency (*U*=12710.50, *p*=0.000) and privacy setting use (*U*=12259.00, *p*=0.000) and lower on social anxiety (*t*=−1.81, *p*=0.072) and perceived stress (*t*=−1.78, *p*=0.076). Nevertheless, only posting frequency (*FDRp*=0.000) and privacy setting use (*FDRp*=0.000) differed significantly between Time Limit user and nonuser groups after applying FDR correction. Also, we found no significant differences in terms of age, gender, life change experiences, self-esteem, self-monitoring, emotional stability, or loneliness between Time Limit users and nonusers.

#### Differences in Social Characteristics

Supplementary Table 3 presents the independent sample *t*-test statistics and FDR correction for the social network characteristics. The results revealed that Time Limit nonusers scored significantly higher in terms of audience size (*t*=−2.68, *p*=0.008) compared with users, after the correction procedure, the FDR-adjusted value of *p* was 0.032. Meanwhile, there were no significant differences in audience diversity between Time Limit users and nonusers.

#### Supplemental Analysis

To further detect the influencing factors of Time Limit setting adoption, we conducted a binary logistic regression analysis by setting Time Limit setting use as a dependent variable (nonuser=0, user=1). As suggested by Meng et al. (2015), to build upon the results of the previous analysis, we only incorporate factors of personal and social characteristics with significant differences into the regression model. Also, we conducted FDR correction. As shown in the Supplementary Table 4, the results show the positive effects of posting frequency (*B*=0.30, *p*=0.002, Exp(*B*)=1.35) and privacy setting use (*B*=0.78, *p*=0.000, Exp(*B*)=2.17) on Time Limit setting use, suggesting that individuals who post frequently and use other privacy settings could be more likely to employ the Time Limit setting. The negative effect of audience size on Time Limit setting use (*B*=−0.46, *p*=0.000, Exp(*B*)=0.63) is also demonstrated, indicating that individuals with large audience sizes would be less likely to apply the Time Limit setting. The above results remain significant after the FDR correction, and the supplemental analysis results support part of our prior findings.

### Differences Between Low-, Medium, and High-Ephemerality User Groups

#### Differences in Personal Characteristics

To examine whether the user groups that had opted for low, medium, or high degrees of ephemerality differed from each other in terms of demographics, personality traits, psychological factors, and previous behavior patterns, we conducted an ANOVA with the personal characteristics as dependent variables and the grouping variable (low, medium, or high) as the factor. No violation of the assumption of variance homogeneity was found, such as the nonsignificant result of Levene’s statistics (except for self-esteem: *p*=0.023 and age: *p*=0.010), for which the Welch F test of robust and asymptotic distribution was adopted (Timmermans et al., 2018). Comparisons between genders were done using the Person chi-square test (Gainsbury et al., 2016). Supplementary Table 5 presents the ANOVA statistics for personal characteristics. The results revealed that there were significant differences in terms of experiences of life changes, self-monitoring, perceived stress, and privacy setting use between users using the Time Limit setting who had opted for low, medium, and high degrees of ephemerality.

#### Differences in Social Characteristics

Supplementary Table 6 presents the ANOVA for social characteristics. The results revealed that there were significant differences in terms of audience size between the user groups with low, medium, and high degrees of ephemerality, but there were no significant differences in terms of audience diversity.

#### *Post hoc* Tests

For variables that differed significantly between time limit user groups with different degrees of ephemerality, we continued to conduct *post hoc* tests and adopted the FDR correction method to adjust the multiple comparison issue. The results revealed that the low-ephemerality user group scored higher on life change experiences than did the high-ephemerality (Md=0.624, *p*=0.000, *FDRp*=0.000) and medium-ephemerality (Md=0.540, *p*=0.000, *FDRp*=0.000) user groups. Also, the low-ephemerality group scored higher on self-monitoring than did the high-ephemerality (Md=0.247, *p*=0.006, *FDRp*=0.012) and medium-ephemerality (Md=0.192, *p*=0.033, *FDRp*=0.033) groups. As for perceived stress, the high-ephemerality user group had higher levels of perceived stress than did the medium (Md=0.150, *p*=0.092, *FDRp*=0.092) and low (Md=0.225, *p*=0.025, *FDRp*=0.050) groups. Meanwhile, the low-ephemerality user group scored higher on posting frequency than did the high-ephemerality group (Z=−2.820, *p*=0.005, *FDRp*=0.010), but scored lower compared with the medium-ephemerality group (Z=−2.109, *p*=0.035, *FDRp*=0.035). The high-ephemerality user group also had smaller audience sizes than both the medium-ephemerality (Md=−0.279, *p*=0.094, *FDRp*=0.094) and low-ephemerality (Md=−0.413, *p*=0.028, *FDRp*=0.056) groups. However, we found no differences in privacy setting use between user groups with different degrees of ephemerality.

## Discussion

This exploratory study examined how users of the Time Limit setting differ from nonusers in terms of personal and social characteristics. We also investigated the differences between user groups with different levels of ephemerality. Our findings indicate significant differences in terms of previous behavior patterns (i.e., posting frequency and privacy setting use) and social characteristics (i.e., audience size) between the Time Limit users and nonusers. Moreover, we also found some differences in personal and social characteristics between user groups with low, medium, and high degrees of ephemerality.

### Differences Between Time Limit Users and Nonusers

In terms of personal characteristics, our results indicate that users using Time Limit setting differ significantly from nonusers in terms of previous behavior patterns. Specifically, users using Time Limit setting reported higher levels of posting frequency and privacy setting use (i.e., the use of tags) compared with nonusers. This result is consistent with previous studies, which indicate that individuals who frequently use social media are more likely to use privacy settings (Stern and Salb, 2015). Moreover, individuals who use the tags feature may be concerned that some audiences who are blocked from viewing their content might nonetheless learn about certain posts from common friends, resulting in interpersonal conflicts or embarrassment (Choi et al., 2015). These concerns may push them to use the Time Limit feature to hide certain posts and destroy evidence of their blocking behaviors on social media. This finding supports Li et al.’s (2018) study concerning that concerns of using tags may facilitate users to employ the Time Limit setting.

Further, the results of education, perceived stress, and social anxiety did not survive FDR correction, suggesting there were no differences between Time Limit users and nonusers regarding these factors. However, minimizing the type I error increases the type II error (Rothman, 1990), so these results need to be interpreted with caution. In terms of education, our result is contrary to previous studies which highlight education is an important factor of innovation adoption (Litt, 2013; Meng et al., 2015) and social media use (Smith et al., 2011; Bogg, 2017). This may be because that using the Time Limit setting does not need much knowledge, resulting in no differences are found between users and nonusers regarding education. Likewise, we found no differences between Time Limit users and nonusers in terms of perceived stress and social anxiety. However, previous studies indicate the ephemerality of content gives users more control over their information (Morlok et al., 2017) and in turn mitigates their concerns of self-presentation and impression management (Bayer et al., 2016; Choi et al., 2020). One possible explanation is that we fail to capture participants’ actual psychological state. Due to social desirability (Krumpal, 2013; Larson, 2019), participants may refuse to report their perceived stress and social anxiety.

In addition, no significant group differences were found for age, gender, or the experience of life changes. Similarly, studies on social media use have reported inconsistent findings regarding users’ demographics (Mohamed and Ahmad, 2012; Chang and Heo, 2014; Li, 2014). In particular, our results are contrary to the findings in Brandtzæg et al.’s (2010) study which indicated that younger adults apply more privacy settings than do older adults. The nonsignificant results may be because that old adults account for a very small percentage in our sample. Also, we found no differences between Time Limit users and nonusers in terms of personality traits regarding self-esteem, self-monitoring, or emotional stability. One possible explanation for this could be the failure of the measures we used to capture individuals’ true traits; for example, only two items from a subscale of the Big Five were used to measure emotional stability. We also did not find any difference in loneliness between Time Limit users and nonusers. One possible explanation is that individuals who experience greater loneliness may use the Time Limit setting to maintain an air of mystery to protect themselves, due to the lack of security sense (Cacioppo and Hawkley, 2009), and persons low in loneliness may use this setting for impression management (Sheldon, 2012; Ljepava et al., 2013).

In terms of social characteristics, users using Time Limit setting scored lower than nonusers on audience size, suggesting that individuals with a larger audience size are less likely to use the Time Limit setting. Studies have claimed that users with large audiences post frequently to maintain interpersonal ties and enhance their social capital (Chang and Heo, 2014; Lankton et al., 2017), thereby compelling them to self-censor their posted content and focus on impression management (Vitak, 2012). Accordingly, individuals with large audiences use past posts to exhibit their long-term identities, thereby making it unnecessary for them to use the Time Limit setting. This finding is also consistent with prior research which shows individuals with large audiences tend to share long-term content to interact with audiences and in turn improve the use of gratification (Wakefield and Bennett, 2018). Moreover, we did not find any difference in audience diversity between Time Limit users and nonusers. This may be because individuals with diverse audiences utilize an alternative strategy regarding strictly manage their audiences (e.g., sharing work links with leaders and colleagues, disclosing personal information with family, and discussing gossip with friends) instead of using the Time Limit setting to achieve impression management (Zheng and Zhao, 2020).

### Differences Between Time Limit User Groups With Different Degrees of Ephemerality

Our results showed some variance in personal characteristics (i.e., life change experiences, self-monitoring, perceived stress, posting frequency, and privacy setting use) and social characteristics (i.e., audience size) between the low-, medium-, and high-ephemerality user groups. The user group with a low degree of ephemerality (i.e., those who had chosen the 6-month option) had more experiences of life changes than the user groups with medium (i.e., those who had chosen the 1-month option) and high (i.e., those who had chosen the 3-day option) degrees of ephemerality. These findings suggest that individuals who experience more life changes may opt for lower degrees of ephemerality. One possible explanation is that individuals who often experience life changes may opt not to post content frequently to maintain consistency in their self-presentation (Ayalon and Toch, 2017). As such, they do not need to choose a more restrictive setting. This finding supports prior ephemerality research revealing that ephemeral content in social media helps users manage their evolving self-presentation or identities (Huang et al., 2020; Luria and Foulds, 2021). Also, personal information management practices indicate that life changes could affect one’s information management strategies (Whittaker and Massey, 2020).

In terms of self-monitoring, our results showed that the users in the low-ephemerality group had higher levels of self-monitoring than did the users in both the medium- and high-ephemerality groups. These results support the notion that individuals who engage in self-monitoring care more about how others evaluate their past posts (Litt and Hargittai, 2014; Zhang et al., 2021); the Time Limit setting may thus help them to manage their future audience. Individuals who self-monitor may also be effective at adjusting their behaviors to align with social norms (Lankton et al., 2017), suggesting that they are more likely to self-censor when they post content to avoid interpersonal uncertainty; this may lead them to prefer a low rather than a medium or high degree of ephemerality. We also found that users using Time Limit setting in the high-ephemerality group had higher levels of perceived stress than did users in both the medium and low-ephemerality groups. One possible explanation is that individuals with higher levels of perceived stress do not have enough energy to manage their past posts, so they choose the more restrictive setting to hide old content to avoid interpersonal uncertainty in the future (Huang et al., 2020). These findings highlight the important role of perceived stress in social media use which was stated in prior studies (Bevan et al., 2014; Wendorf and Yang, 2015).

In terms of posting frequency, we found that users using Time Limit setting in the medium-ephemerality group posted more frequently than users in the low-ephemerality group; users in the low-ephemerality group posted more frequently than users in the high-ephemerality group. These findings suggest that individuals who post frequently are more likely to choose the 1- or 6-month option rather than the 3-day option. This may be because individuals who post frequently are often active social media users, and they might be concerned that using the restrictive setting (i.e., 3-day option) may leave a negative impression on others (e.g., isolated, not friendly, or aloof; Huang et al., 2020). Thus, active users could not employ the restricted Time Limit setting to avoid damaging their social capital. This finding indirectly supports the point that using Time Limit setting may undermine social interactions especially the use of restrictive ones (Li et al., 2018). Meanwhile, in terms of social characteristics, we found that users using Time Limit setting with larger audiences were more likely to choose low or medium-ephemerality options rather than the high option. Individuals with larger audiences may often utilize the “lowest common denominator” strategies regarding only post information that is suitable for everyone (Hogan, 2010), to deal with posting content to large and diverse audiences. As such, they do not need to utilize the overly restive Time Limit setting.

## Implications and Limitations

### Theoretical Implications

Our findings have several theoretical implications. First, we extend privacy setting research by discussing a specific feature (i.e., Time Limit setting), while previous studies mainly focused on general privacy settings (Litt, 2013; Stern and Salb, 2015). Considering specific characteristics of a popular privacy setting could deepen our understanding of users’ attitudes and behavior when using social media. Moreover, our work enriches the literature on ephemerality in social media by exploring differences between user and non-user of an ephemerality-related design, while limited previous studies on ephemerality mainly examined how ephemeral content impacted users’ social and emotional experiences through the in-depth interviews (Bayer et al., 2016; Xu et al., 2016; Huang et al., 2020). By investigating how personal and social characteristics varied between users and nonusers of an ephemerality setting, our work adds new knowledge about the availability of ephemerality features in social media.

Second, we highlight the critical role of personal characteristics in ephemerality setting use, by systematically exploring how Time Limit users differ from nonusers regarding demographics, personality traits, psychological factors, and previous behavior patterns. Although several ephemerality studies indicated individual characteristics (e.g., life changes, maturity) would impact ephemeral content engagement in social media (Li et al., 2018; Luria and Foulds, 2021), as well as ephemerality feature could mitigate users’ concerns (Bayer et al., 2016; Choi et al., 2020), these studies are fragment and most of them based on the qualitative method. Our work extends these prior studies as we empirically verified that individuals with higher level of posting frequency and privacy setting use would be more likely to employ the Time Limit setting.

Third, our work enriches the knowledge about how characteristics of social networks impact ephemerality setting use, by investigating differences in audience diversity and audience size between Time Limit users and nonusers. Although one study claimed changes in users’ social networks could encourage them to engage in ephemeral content (Huang et al., 2020), there is a lack of theoretical understanding about what specific factors of social networks would exert these effects. We demonstrated that individuals with larger audience size would be less likely to employ the Time Limit setting, implying the negative impact of ephemerality feature on social interactions, which enrich the literature on negative consequences (e.g., feeling of loss and undermining social relationships) of using ephemerality in social media (Bayer et al., 2016; Cavalcanti et al., 2017).

Finally, we shed new light on studying ephemerality in social media by detecting the differences between user groups that opted for different levels of ephemerality. While one study described users’ perception of short-term and long-term ephemerality and suggested ephemerality could support users’ evolving identities through an 8-day qualitative diary study (Luria and Foulds, 2021), it failed to explain why individuals choose different levels of ephemerality in social media. Our work extends prior research by indicating that factors regarding life change experiences, self-monitoring, perceived stress, posting frequency, and audience size may impact the selection of different degrees of ephemerality. The present study also responds to the call for more detailed research on exploring different degrees of ephemerality in social media (Xu et al., 2016; Huang et al., 2020).

### Practical Implications

This study provides useful insights for social media practitioners. In particular, our findings can guide practitioners in designing more effective ephemerality settings on social media platforms. Our findings suggest that posting frequency, privacy setting use, and audience size are significant predictors of whether individuals use or do not use the Time Limit setting on WeChat Moments. Consideration of these influencing factors can help practitioners improve ephemerality features on social media platforms.

Specifically, since our findings show that individuals’ other privacy setting use (i.e., the use of tags) could impact how they employ Time Limit setting, platforms could inform users of more details about differences and links between current ephemerality setting and other privacy tools. This is because the final outcome of ephemerality setting use is associated with the implementation of other privacy management strategies. Moreover, individuals with larger audiences preferred not to use the Time Limit setting, indicating that the costs of using this setting (e.g., the loss of the ability to display valuable past posts to enable social interactions) may be a dissuading factor. A possible response from practitioners to this information may be to make ephemerality settings more flexible, such as by enabling a user to toggle ephemerality settings for individual posts rather than for one’s entire posting history or by enabling a user to toggle ephemerality settings for certain audiences rather than one’s entire audience. Also, we found some variance in personal and social characteristics between Time Limit user groups that opted for different degrees of ephemerality, suggesting that individuals have different ephemerality needs on social media platforms. Building on this insight, practitioners may opt to allow users to set a specific time span (e.g., 1day) for their past posts to remain visible to their audiences, rather than limiting their time span options.

### Limitations and Future Research

This study is limited in a few ways. First, given that social media platforms increasingly provide users with the option to make their posts ephemeral, focusing only on the Time Limit setting in WeChat Moments to explore our research questions limited the generalizability of our findings (Montag et al., 2018). Future studies could investigate the ephemerality settings on other platforms such as Snapchat or Instagram. Also, cross-cultural studies are encouraged to examine the differences in ephemerality setting usage across different cultures.

Second, the majority of our respondents were company employees, and individuals in different occupations may have different perceptions of the Time Limit feature. Future research could investigate a more representative sample to confirm our findings. Moreover, most of our respondents are young people, future studies could explore how old adults use the Time Limit setting. For Research Question 2, we worked with small subgroups, indicating that our data may not have had enough statistical power to allow for the identification of significant differences regarding personal and social characteristics. Future research could explore this issue further with larger samples. Also, cross-cultural studies are encouraged to explore differences in users’ perceptions of using ephemerality settings on social media platforms.

Third, our data were collected through self-reports, which may have limited our understanding of users’ actual personal characteristics. This may explain why few significant differences in personality traits and psychological factors were found between Time Limit users and nonusers. Further research could use experience sampling or secondary data to more effectively capture users’ personal characteristics.

Fourth, we only compared personal and social characteristics between Time Limit users and nonusers, future studies could examine individuals’ other perceptions (e.g., the benefits and costs of using ephemerality settings, social influence, or interpersonal relationships). In particular, regarding factors of privacy setting use, we only explored how using tags influence users’ Time Limit setting usage, but failed to consider how the effects of other privacy management strategies (e.g., using multiple accounts, only disclosing non-sensitive information). Thus, future studies could further elaborate these ideas. Meanwhile, this study only considered three factors of personality traits and found few significant differences for these variables. This may be due to the measure we used, and future research could explore the use of more effective measurements (e.g., the Big Five) and could consider other personality traits (e.g., narcissism and shyness; Scott et al., 2018; Yu et al., 2020). We also encourage researchers to investigate how motives may vary between Time Limit user groups with different degrees of ephemerality. In addition, we did not ask participants how long and how often they used the Time Limit feature; whether a user is an early or late adopter of the feature may be relevant as well.

## Conclusion

Given that most individuals are concerned about the long-term visibility of their past posts, it is becoming more common for social media platforms to provide users with the option to make their content ephemeral. Nonetheless, the number of studies that explore ephemerality settings on social media is limited. Using the Time Limit feature on WeChat Moments as an example, this study is the first to provide empirical insight into the differences between users and nonusers of ephemerality settings. Our findings indicate that compared with nonusers of the feature, users using Time Limit setting post content more frequently, use the Friend Lists feature, and have smaller audiences. Meanwhile, our findings indicate that among Time Limit users, those who have experienced more life changes have higher levels of self-monitoring and less perceived stress, post content more frequently, and have larger audiences are more likely to opt for a low degree of ephemerality (i.e., the 6-month option) rather than a medium (i.e., the 1-month option) or high (i.e., the 3-day option) degree. Customizable settings on social media platforms should be improved over time to support users’ evolving attitudes and behaviors. Thus, it is essential to explore this new ephemerality setting further to provide insights that can facilitate the development of social media platforms.

## Data Availability Statement

The original contributions presented in the study are included in the article/Supplementary Material, further inquiries can be directed to the corresponding author.

## Author Contributions

YZ contributed to the idea of the study and wrote the manuscript. HW performed the data analyses and wrote the manuscript. CL contributed to the analysis and manuscript preparation. SC performed the data collection and helped with the analysis part. All authors contributed to the article and approved the submitted version.

## Funding

This work was supported by Fundamental Research Funds for the Central Universities (No. JBK2101026).

## Conflict of Interest

The authors declare that the research was conducted in the absence of any commercial or financial relationships that could be construed as a potential conflict of interest.

## Publisher’s Note

All claims expressed in this article are solely those of the authors and do not necessarily represent those of their affiliated organizations, or those of the publisher, the editors and the reviewers. Any product that may be evaluated in this article, or claim that may be made by its manufacturer, is not guaranteed or endorsed by the publisher.

## Footnotes

^1^ https://www.sojump.com
