# Supplementary material for: Ephemerality in Social Media: Unpacking the Personal and Social Characteristics of Time Limit Users on WeChat Moments
Source: Front Psychol. 2021 Sep 6;12:712440. doi: 10.3389/fpsyg.2021.712440 (PMC8451155; doi:10.3389/fpsyg.2021.712440)

Supplementary Material

**Table 1 Descriptive statistics and correlations (*N* = 390)**

| **Variables** | **Min** | **Max** | **Mean** | ***SD*** | **1** | **2** | **3** | **4** | **5** | **6** | **7** | **8** | **9** | **10** | **11** |
| --- | --- | --- | --- | --- | --- | --- | --- | --- | --- | --- | --- | --- | --- | --- | --- |
| ***Demographics*** |  |  |  |  |  |  |  |  |  |  |  |  |  |  |  |
| 1. Life change experiences | 1.00 | 5.00 | 3.46 | 1.00 | 1 |  |  |  |  |  |  |  |  |  |  |
| ***Personality traits*** |  |  |  |  |  |  |  |  |  |  |  |  |  |  |  |
| 2. Self-esteem | 1.78 | 5.00 | 4.05 | 0.65 | -0.05 | 1 |  |  |  |  |  |  |  |  |  |
| 3. Self-monitoring | 1.57 | 5.00 | 3.54 | 0.58 | 0.04 | 0.63** | 1 |  |  |  |  |  |  |  |  |
| 4. Emotional stability | 1.00 | 4.50 | 2.17 | 0.84 | 0.08 | -0.70** | -0.50** | 1 |  |  |  |  |  |  |  |
| ***Psychological factors*** |  |  |  |  |  |  |  |  |  |  |  |  |  |  |  |
| 5. Social anxiety | 1.00 | 4.50 | 2.30 | 0.80 | 0.03 | -0.75** | -0.72** | 0.67** | 1 |  |  |  |  |  |  |
| 6. Perceived stress | 1.00 | 4.29 | 2.25 | 0.64 | 0.02 | -0.82** | -0.71** | 0.73** | 0.82** | 1 |  |  |  |  |  |
| 7. Loneliness | 1.10 | 4.60 | 2.30 | 0.67 | 0.11* | -0.72** | -0.55** | 0.64** | 0.75** | 0.77** | 1 |  |  |  |  |
| ***Previous behavior patterns*** |  |  |  |  |  |  |  |  |  |  |  |  |  |  |  |
| 8. Posting frequency | 1.00 | 5.00 | 3.06 | 1.40 | 0.11* | 0.35** | 0.35** | -0.28** | -0.36** | -0.30** | -0.21** | 1 |  |  |  |
| 9. Privacy setting use | 1.00 | 3.00 | 2.18 | 0.67 | 0.15** | 0.12* | 0.13** | -0.11* | -0.11* | -0.15** | -0.08 | 0.32** | 1 |  |  |
| ***Social characteristics*** |  |  |  |  |  |  |  |  |  |  |  |  |  |  |  |
| 10. Audience size | 1.00 | 5.00 | 2.79 | 1.25 | 0.21** | 0.18** | 0.20** | -0.11* | -0.22** | -0.19** | -0.15** | 0.30** | 0.16** | 1 |  |
| 11. Audience diversity | 1.00 | 11.00 | 6.81 | 1.88 | 0.14** | 0.14** | 0.13* | -0.10 | -0.16** | -0.11* | -0.14** | 0.11* | 0.01 | 0.36** | 1 |

*Note*. ^**^ *p<0.01;* ^*^ *p<0.05*


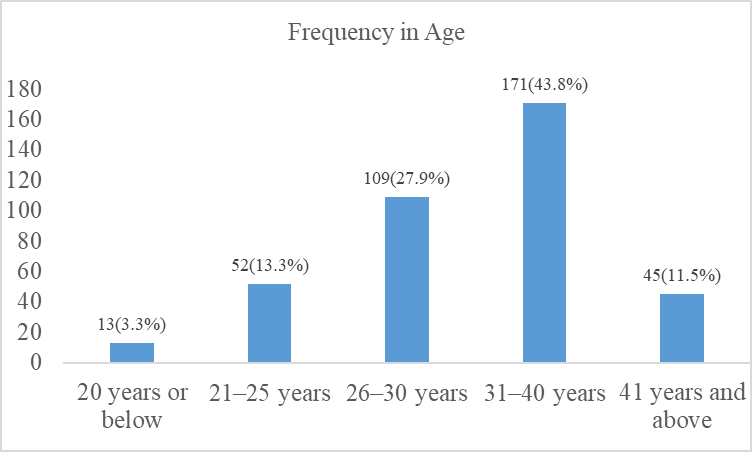

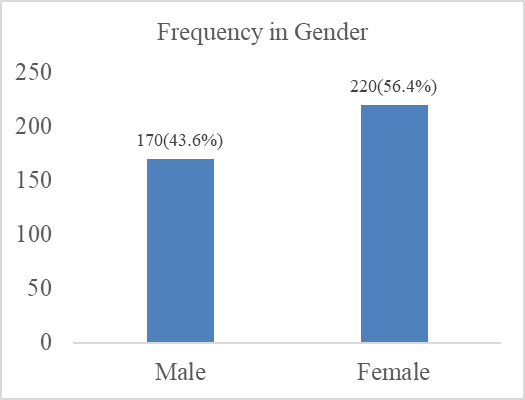


**Figure 1 Frequency in Age**  **Figure 2 Frequency in Gender**


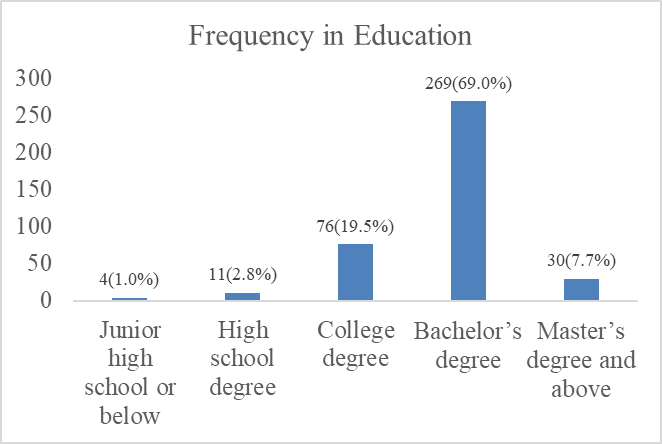
 **Figure 3 Frequency in Education**

**Table 2 Statistical comparison of personal characteristics of Time Limit users and nonusers**

|  | Time limit users  (n = 263) | |  | Time limit nonusers  (n = 127) | |  |  | | |
| --- | --- | --- | --- | --- | --- | --- | --- | --- | --- |
|  | Mean | SD |  | Mean | SD |  | *t/U/χ^2^* | *p* | *FDRp* |
| ***Demographics*** |  |  |  |  |  |  |  |  |  |
| Age | 3.49 | 0.94 |  | 3.42 | 1.05 |  | 0.73 | 0.465 | 0.592 |
| Gender^b^ | 1.57 | 0.50 |  | 1.56 | 0.50 |  | 0.02 | 0.889 | 0.889 |
| Education | 3.84 | 0.55 |  | 3.69 | 0.84 |  | 1.85 | 0.066 | 0.231 |
| Life change experiences | 3.49 | 0.97 |  | 3.42 | 1.06 |  | 0.63 | 0.529 | 0.617 |
| ***Personality traits*** |  |  |  |  |  |  |  |  |  |
| Self-esteem | 4.07 | 0.62 |  | 4.00 | 0.70 |  | 1.00 | 0.277 | 0.388 |
| Self-monitoring | 3.57 | 0.57 |  | 3.48 | 0.59 |  | 1.47 | 0.144 | 0.288 |
| Emotional stability | 2.16 | 0.84 |  | 2.20 | 0.86 |  | -0.41 | 0.684 | 0.737 |
| ***Psychological factors*** |  |  |  |  |  |  |  |  |  |
| Social anxiety | 2.25 | 0.79 |  | 2.40 | 0.80 |  | -1.81 | 0.072 | 0.202 |
| Perceived stress | 2.21 | 0.63 |  | 2.33 | 0.68 |  | -1.78 | 0.076 | 0.177 |
| Loneliness | 2.26 | 0.68 |  | 2.37 | 0.67 |  | -1.43 | 0.153 | 0.268 |
| ***Previous behavior patterns*** |  |  |  |  |  |  |  |  |  |
| Posting frequency ^a^ | 3.25 | 1.33 |  | 2.66 | 1.46 |  | 12710.50 | 0.000 | 0.000 |
| Privacy setting use ^a^ | 2.30 | 0.61 |  | 1.94 | 0.72 |  | 12259.00 | 0.000 | 0.000 |

*Note*: ^a^ indicates that Mann-Whitney U tests were used. ^b^ indicates that Person chi-square tests were used.

**Table 3 Statistical comparison of social characteristics of Time Limit users and nonusers**

|  | Time limit users  (n = 263) | |  | Time limit nonusers  (n = 127) | |  |  | | |
| --- | --- | --- | --- | --- | --- | --- | --- | --- | --- |
|  | Mean | SD |  | Mean | SD |  | *t* | *p* | *FDRp* |
| ***Social characteristics*** |  |  |  |  |  |  |  |  |  |
| Audience size | 2.67 | 1.18 |  | 3.03 | 1.36 |  | -2.68 | 0.008 | 0.037 |
| Audience diversity | 6.73 | 1.81 |  | 6.96 | 2.01 |  | -1.14 | 0.257 | 0.400 |

**Table 4 Logistic regression analysis of Time Limit setting use**

| Independent var. | *B* | S.E. | *p* | *FDRp* | Exp(*B*) |
| --- | --- | --- | --- | --- | --- |
| Education | 0.26 | 0.17 | 0.138 | 0.207 | 1.29 |
| Society anxiety | -0.17 | 0.26 | 0.509 | 0.611 | 0.85 |
| Perceived stress | 0.02 | 0.31 | 0.961 | 0.961 | 1.02 |
| Posting frequency | 0.30 | 0.10 | 0.002 | 0.004 | 1.35 |
| Privacy setting use | 0.78 | 0.19 | 0.000 | 0.000 | 2.17 |
| Audience size | -0.46 | 0.10 | 0.000 | 0.000 | 0.63 |

*Note.* N = 390; -2 log likelihood = 437.442;

Nagelkerke R^2^ = .183; percentage correct: 74.4%.

**Table 5 Statistical comparison of personal characteristics of Time Limit user groups with low, medium and high degrees of ephemerality**

|  | High degree  of ephemerality  (*n* = 97) | |  | Medium degree  of ephemerality  (*n* = 101) | |  | Low degree  of ephemerality  (*n* = 65) | |  | ANOVA | |
| --- | --- | --- | --- | --- | --- | --- | --- | --- | --- | --- | --- |
|  | Mean | SD |  | Mean | SD |  | Mean | SD |  | *F/χ^2^* | *p* |
| ***Demographics*** |  |  |  |  |  |  |  |  |  |  |  |
| Age ^c^ | 3.44 | 1.01 |  | 3.61 | 0.80 |  | 3.38 | 1.01 |  | 1.550 | 0.216 |
| Gender ^b^ | 1.64 | 0.48 |  | 1.50 | 0.50 |  | 1.57 | 0.50 |  | 4.188 | 0.126 |
| Education | 3.80 | 0.53 |  | 3.82 | 0.52 |  | 3.94 | 0.61 |  | 1.310 | 0.272 |
| Life change experiences | 3.30 | 1.00 |  | 3.38 | 0.95 |  | 3.92 | 0.84 |  | 9.492 | 0.000 |
| ***Personality traits*** |  |  |  |  |  |  |  |  |  |  |  |
| Self-esteem ^c^ | 3.98 | 0.69 |  | 4.11 | 0.53 |  | 4.16 | 0.62 |  | 1.696 | 0.187 |
| Self-monitoring | 3.48 | 0.61 |  | 3.54 | 0.53 |  | 3.73 | 0.53 |  | 3.969 | 0.020 |
| Emotional stability | 2.20 | 0.89 |  | 2.12 | 0.77 |  | 2.16 | 0.86 |  | 0.183 | 0.833 |
| ***Psychological factors*** |  |  |  |  |  |  |  | |  |  |  |
| Social anxiety | 2.38 | 0.88 |  | 2.20 | 0.72 |  | 2.14 | 0.74 |  | 2.164 | 0.117 |
| Perceived stress | 2.32 | 0.65 |  | 2.17 | 0.59 |  | 2.10 | 0.62 |  | 2.838 | 0.060 |
| Loneliness | 2.37 | 0.69 |  | 2.21 | 0.59 |  | 2.18 | 0.75 |  | 2.023 | 0.134 |
| ***Previous behavior patterns*** |  |  |  |  |  |  |  |  |  |  |  |
| Posting frequency ^b^ | 2.92 | 1.43 |  | 3.47 | 1.25 |  | 3.40 | 1.38 |  | 21.546 | 0.006 |
| Privacy setting use ^b^ | 2.23 | 0.67 |  | 2.34 | 0.60 |  | 2.35 | 0.51 |  | 8.327 | 0.080 |

*Note.* Those who selected the 3-day option were considered to have a high degree of ephemerality; those who had selected

the 1-month option were considered to have a medium degree of ephemerality; those who had selected the 6-month option were

considered to have a low degree of ephemerality.

^b^ indicates that Person chi-square tests were used. ^c^ indicates that the Welch F-test was used.

**Table 6 Statistical comparison of social characteristics of Time Limit user groups with low, medium and high degrees of ephemerality**

|  | High degree  of ephemerality  (n = 97) | |  | Medium degree  of ephemerality  (n = 101) | |  | Low degree  of ephemerality  (n = 65) | |  | ANOVA | |
| --- | --- | --- | --- | --- | --- | --- | --- | --- | --- | --- | --- |
|  | Mean | SD |  | Mean | SD |  | Mean | SD |  | *F* | *p* |
| ***Social characteristics*** |  |  |  |  |  |  |  |  |  |  |  |
| Audience size | 2.46 | 1.21 |  | 2.74 | 1.09 |  | 2.88 | 1.22 |  | 2.726 | 0.067 |
| Audience diversity | 6.65 | 1.79 |  | 6.61 | 1.77 |  | 7.03 | 1.91 |  | 1.198 | 0.303 |

*Note.* Those who had selected the 3-day option were considered to have a high degree of ephemerality; those who had

selected the 1-month option were considered to have a medium degree of ephemerality; those who had selected the 6-month

option were considered to have a low degree of ephemerality.

Appendix A. The Time Limit setting on WeChat Moments


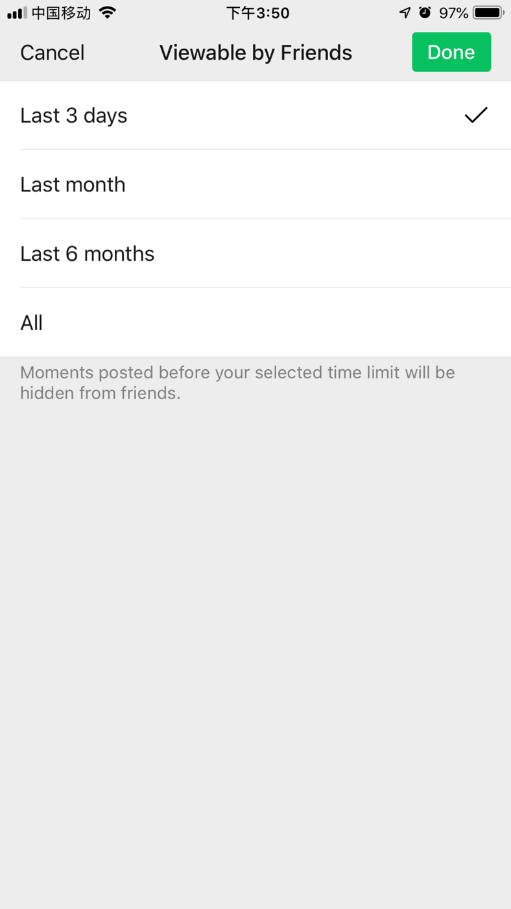


Appendix B. The Time Limit interface on WeChat Moments when not using Time Limit (left) vs. using three-day Time Limit (right)


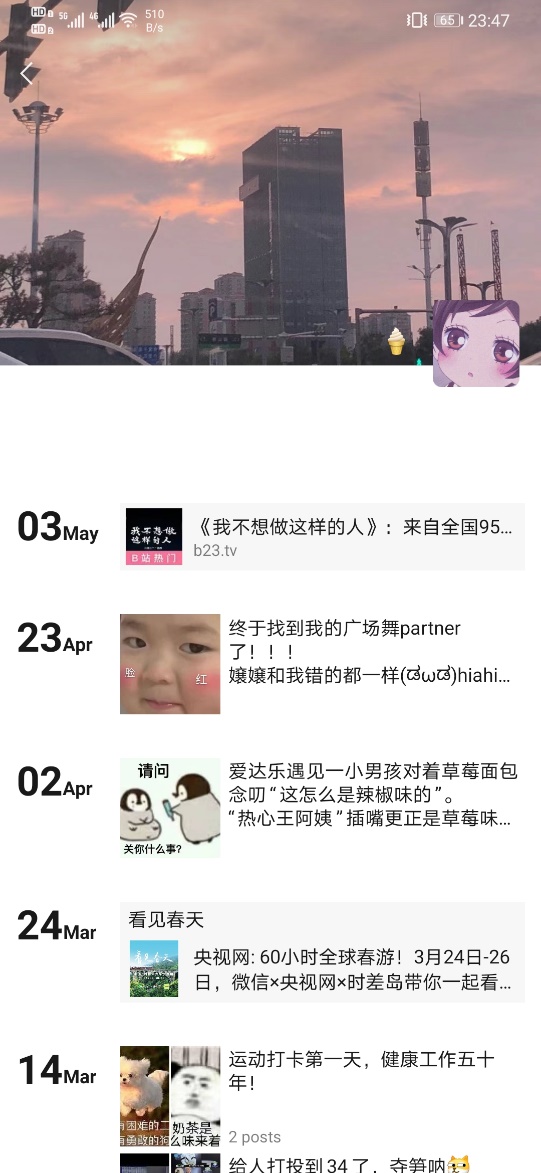

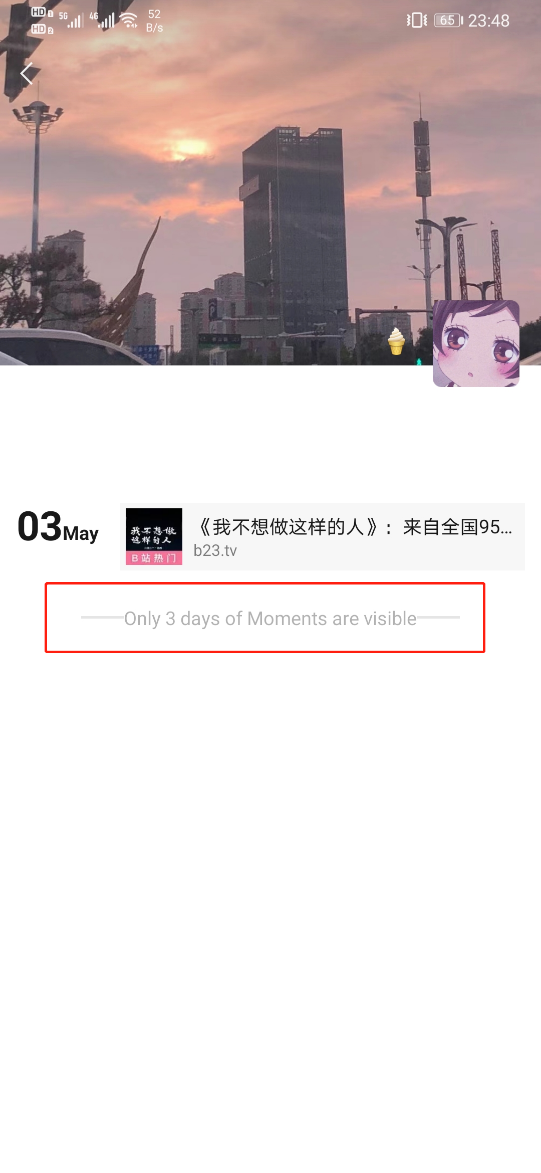


Appendix C. The tags feature on WeChat Moments vs. Friend Lists on Facebook


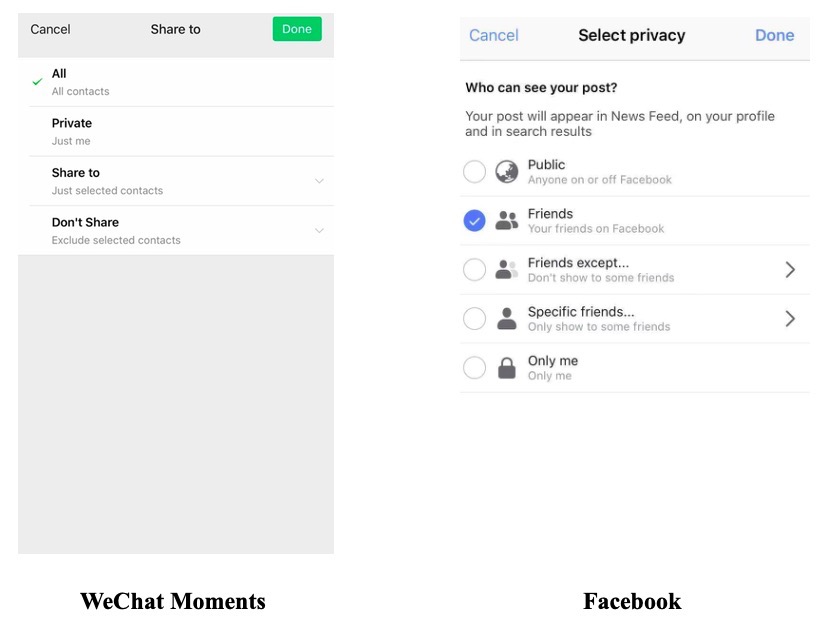

Supplement: Supplementary file 1 [file Data_Sheet_1.docx]
